# Long-term and combined effects of N-[2-(nitrooxy)ethyl]-3-pyridinecarboxamide and fumaric acid on methane production, rumen fermentation, and lactation performance in dairy goats

**DOI:** 10.1186/s40104-021-00645-4

**Published:** 2021-12-06

**Authors:** Zongjun Li, Xinjian Lei, Xiaoxu Chen, Qingyan Yin, Jing Shen, Junhu Yao

**Affiliations:** College of Animal Science and Technology, Northwest A&F University, Yangling, 712100 Shaanxi China

**Keywords:** Bacterial populations, Dairy goat, Fumaric acid, Lactation performance, Methane emissions, N-[2-(nitrooxy)ethyl]-3-pyridinecarboxamide, Rumen fermentation

## Abstract

**Background:**

In recent years, nitrooxy compounds have been identified as promising inhibitors of methanogenesis in ruminants. However, when animals receive a nitrooxy compound, a high portion of the spared hydrogen is eructated as gas, which partly offsets the energy savings of CH_4_ mitigation. The objective of the present study was to evaluate the long-term and combined effects of supplementation with N-[2-(nitrooxy)ethyl]-3-pyridinecarboxamide (NPD), a methanogenesis inhibitor, and fumaric acid (FUM), a hydrogen sink, on enteric CH_4_ production, rumen fermentation, bacterial populations, apparent nutrient digestibility, and lactation performance of dairy goats.

**Results:**

Twenty-four primiparous dairy goats were used in a randomized complete block design with a 2 × 2 factorial arrangement of treatments: supplementation without or with FUM (32 g/d) or NPD (0.5 g/d). All samples were collected every 3 weeks during a 12-week feeding experiment. Both FUM and NPD supplementation persistently inhibited CH_4_ yield (L/kg DMI, by 18.8% and 18.1%, respectively) without negative influence on DMI or apparent nutrient digestibility. When supplemented in combination, no additive CH_4_ suppression was observed. FUM showed greater responses in increasing the molar proportion of propionate when supplemented with NPD than supplemented alone (by 10.2% vs. 4.4%). The rumen microbiota structure in the animals receiving FUM was different from that of the other animals, particularly changed the structure of phylum Firmicutes. Daily milk production and serum total antioxidant capacity were improved by NPD, but the contents of milk fat and protein were decreased, probably due to the bioactivity of absorbed NPD on body metabolism.

**Conclusions:**

Supplementing NPD and FUM in combination is a promising way to persistently inhibit CH_4_ emissions with a higher rumen propionate proportion. However, the side effects of this nitrooxy compound on animals and its residues in animal products need further evaluation before it can be used as an animal feed additive.

## Introduction

Methane (CH_4_) emissions from ruminants not only contribute to anthropogenic greenhouse gas emissions and enlarge the carbon footprint of dairy or beef production [1] but also drain dietary energy (2% to 12% of gross energy (GE)) [2]. Successful CH_4_ mitigation strategies should have persistent efficacy and have no adverse effect on feed degradation, animal health, and productivity [1, 3]. In recent years, nitrooxy (−O − NO_2_) compounds, such as 3-nitrooxypropanol (3-NOP), have been identified as promising methanogenesis inhibitors [4–8] that specifically dock into the active site of methyl-CoM reductase, a key enzyme in the methanogenesis pathway. As a nitrooxy compound, N-[2-(nitrooxy)ethyl]-3-pyridinecarboxamide (NPD) effectively decreased CH_4_ production *in vitro* [8]. When the methanogenesis pathway is inhibited, hydrogen production increased and is eructated as gas (increased by 48- to 100-fold) [6, 8–10]_,_ and hydrogen is also a greenhouse gas with high energy [11, 12]. It suggested that the efficiency of hydrogen capture was lower when CH_4_ production was inhibited. Propionogenesis is the second large hydrogen sink after methanogenesis [13, 14], and it is a more energy-rendering fermentation pathway for animals [14, 15]. Fumaric acid (FUM), a metabolic intermediate of the propionate-forming pathways, has been identified as a promising propionate enhancer and methanogenesis competitor for hydrogen [16, 17]. Therefore, we hypothesized that a combination of NPD and FUM might divert more hydrogen from methanogenesis to propionate synthesis than each inhibitor alone.

Nitrooxy compounds have also been used to treat angina [18]. NPD is a nicotinamide derivative and a balanced vasodilator, which is also called Nicorandil, one of the most effective, healthy and widely used angina drugs, because of its functions as a K^+^_ATP_ channel opener and NO donor [18]. To our knowledge, the side effects of nitrooxy compounds as animal feed additives have been rarely mentioned. The objective of the current study was to evaluate the persistent and combined effects of supplementation with NPD and FUM on CH_4_ suppression, rumen fermentation, rumen bacterial population, apparent nutrient digestibility, serum total antioxidant capacity, and milk performance in lactating dairy goats.

## Methods

All experimental procedures were approved by the Northwest A&F University Animal Care and Use Committee.

### Animals, diets, and experimental design

Twenty-four primiparous Guanzhong dairy goats (113 ± 9 days in milk (DIM), 39 ± 3.8 kg of body weight (BW) at the start of the experiment) were chosen from a dairy goat farm (Shaanxi, China) and blocked into six blocks by DIM, BW, and daily milk production (DMP). Animals within each block were randomly assigned to 1 of 4 dietary treatments: control (CON), a basal diet without any additives; basal diet supplemented with FUM (Aladdin^®^, Shanghai, China) at 34 g/d; basal diet supplemented with NPD (J&K Scientific^®^, Beijing, China) at 0.5 g/d; and the basal diet supplemented with both FUM (34 g/d) and NPD (0.5 g/d). The supply dose of FUM was based on the data published previously [17], while that of NPD was based on a previous 3-NOP study [6] and a mice study [19]. The ration was fed as total mixed ration (TMR, Table 1) twice daily at 0730 and 1730 h and was provided individually at 105% of the expected feed intake (as-fed basis) based on the amounts of feed offered and refused from the previous day. The FUM and/or NPD was top-dressed on one-quarter of the offered TMR that was fed first to ensure complete intake. All goats were individually housed in 24 tie-stalls in a barn and had free access to water. The goats were milked twice daily at feeding. The milk produced by the goats receiving NPD was discarded.

**Table 1:** Ingredients and chemical composition of the experimental diet

Item	%
Ingredients	
Corn silage	21.3
Alfalfa hay	30.8
Ground corn	22.9
Soybean meal	6.6
Cottonseed meal	5.0
Corn germ meal	3.2
Wheat bran	8.2
CaHPO_4_	0.5
CaCO_3_	0.5
NaHCO_3_	0.3
Salt	0.5
Vitamin-mineral premix^a^	0.2
Chemical composition, % of DM	
DM	47.0
EE	4.1
Ash	6.7
CP	18.6
NDF	36.1
ADF	20.4

^a^Vitamin-mineral premix (per kg): 600 mg of Mn, 950 mg of Zn, 430 mg of Fe, 650 mg of Cu, 30 mg of Se, 45 mg of I, 20 mg of Co, 450 mg of nicotinic acid, 800 mg of vitamin E, 45 kIU of vitamin D, and 120 kIU of vitamin A

The feeding experiment lasted 12 weeks, and all samples were collected or measured at weeks 3, 6, 9, and 12. The six blocks of goats were divided into 3 groups by DIM, and the feeding experiment started in a staggered manner for the 3 groups with a 7-d interval so that gas emissions from each group could be measured in turn using the four indoor environmental chambers (each 7.4 m × 4.2 m × 2.7 m) available. Two goats within the same treatment were placed in one chamber and were separated by placing each in a metabolic cage (1.5 m × 1.0 m × 1.5 m). The goats were moved from barn to chambers one day before sample collection and measurements, and no stress responses were observed because they had already adapted to the chambers before the feeding experiment. On d 1–4 during each sample collecting week, total-tract digestibility of dietary nutrients, milk composition, and CH_4_ emissions were measured simultaneously, and the samples of blood and rumen content were collected on d 4–5 and d 5–6, respectively.

### Measuring CH_4_ emissions and milk performance

Gas emissions in the environmental chambers were measured as previously described [17, 20] with minor changes. Briefly, the daily (22 h; 08:30 to 17:30 and 18:30 to 07:30) gas emissions from each chamber were measured in 3 consecutive days. During the gas measurement, the internal temperature of the chambers was maintained to be the same as the ambient temperature outside the building. The air inside each chamber was mixed for 30 s every 10 min by 4 draft fans. The gases from the four chambers and external environment were continuously and constantly pumped at a rate of 4 L/min by 5 exhaust fans. The pumped gases were analyzed sequentially by an FID sensor (Thermo Scientific 55i, USA), 12 min for each in every 60 min.

The daily CH_4_ production was calculated as follows:

$$ {\mathrm{CH}}_4\mathrm{production}\ \left(\mathrm{L}/\mathrm{d}\right)=\Sigma\ \left[\left({C}_i-{C}_{i- 1}\right)\times {\mathrm{V}}_{\mathrm{c}}+{\mathrm{V}}_{\mathrm{f}}\times \left({C}_i-{CO}_i\right)\right]/1000 $$

Where *C*_*i*_ = the CH_4_ concentration (mL/m^3^) of the internal chamber at the *i* 60-min; *CO*_*i*_ = the CH_4_ concentration of external environment at the *i* 60-min; V_c_ = the chamber volume (83.9 m^3^); and V_f_ = the gas volume pumped from each chamber over each 60-min measurement (0.24 m^3^).

During each of the two one-hour no-measurement periods, the chamber doors were opened, and the fresh-air exchange fans were running to exchange fresh air. Meanwhile, the goats were milked and fed, and the samples of milk and orts of individual goats were collected. During these 3 consecutive days, the morning and evening milk production of each goat were recorded and mixed, and 50 mL was subsampled and stored at 4 °C until analysis for milk composition. Milk samples were analyzed for fat, protein, lactose, and milk urea nitrogen (MUN) using an infrared milk analyzer (MilkoScan FT 120, FOSS, Hillerød, Denmark) within 24 h. Fat corrected milk (FCM) was calculated according to NRC (2001) [21]: milk fat yield (kg/d) × 16.216 + milk yield (kg/d) × 0.4324, and net energy for lactation (NEL, Mcal/d) = milk yield (kg/d) × ((0.0929 × percent fat) + (0.0563 × percent true protein) + (0.0395 × percent lactose)).

### Apparent total tract digestibility and energy balance

The apparent total tract digestibility and energy balance of each goat were estimated by daily total collection of feces and urine from d 1–4 during experimental weeks 3 and 9. All refusals and feces of individual goats were dried at 55 °C for 72 h in forced air ovens to a constant weight and subsample (about 100 g, wool removed) was ground through a 1-mm screen for further analysis. Urine was collected through a funnel into buckets and acidified by adding 100 mL of 10% (vol/vol) sulfuric acid to prevent microbial degradation and the loss of volatile ammonia-N. These samples were determined the contents of dry matter (DM), ash, and crude protein (CP) [22]. Neutral detergent fiber (NDF) and acid detergent fiber (ADF) contents were measured using the filter bag method with sodium sulfite and heat-stable α-amylase (Ankom^®^ A200I fiber analyser, ANKOM Technology, Macedon, NY, USA). The BW was recorded twice daily after milking. The gross energy (GE) content of the samples was analyzed in an automatic adiabatic bomb calorimeter (model 1600 Parr Instrument Co., Moline, IL, USA). Digestible energy (DE) was calculated as the difference between energy intake and fecal energy; the energy lost as CH_4_ was calculated as the CH_4_ emitted in L/day × 39.54 kJ/L; metabolizable energy (ME) was the difference between DE and the sum of the energy in urine and CH_4_.

### Collection and analysis of blood samples

Blood samples were collected from an external jugular vein into two 10-mL blood tubes before the morning feeding on two consecutive days in each sample collection week. The sample in the tube was allowed to clot at room temperature for 30 min and centrifuged (3000 × *g*, 15 min) thereafter to obtain serum, which was stored at − 80 °C for later analysis. Serum malondialdehyde (MDA) concentration, total antioxidant capacity (T-AOC), and the activities of serum glutathione peroxidase (GSH-Px) and superoxide dismutase (SOD) were analyzed using respective commercial kits (Jiancheng Bioengineering Institute, Nanjing, China).

### Collection and analysis of ruminal samples

Ruminal content samples were collected using an oral tube and a hand vacuum pump at 6 h after the morning feeding in 2 consecutive days in each sample collection week. To minimize saliva contamination, approximately 50 mL of ruminal fluid was discarded before sample collection. Ruminal pH was measured immediately after sampling. Rumen fluid was subsampled for analysis of volatile fatty acids (VFA, 5 mL with 1 mL of 25% metaphosphoric acid added), organic acids (5 mL), and microbiota (45 mL), and then stored at − 80 °C until analysis.

Ruminal VFA concentration was determined using gas chromatography (Agilent Technologies 7820A GC system, Palo Alto, CA, USA) as described by Li et al. [23]. Ruminal organic acid (fumarate, succinate, and lactate) concentration was determined using an Agilent 1260 high-performance liquid chromatography system as done in previous studies [24, 25].

### Bacterial community analysis

Rumen content samples of each goat from each week were freeze-dried and mixed. Microbial genomic DNA was extracted using a QIAamp DNA Stool Mini Kit (Qiagen, Hilden, Germany) according to the manufacturer’s instruction. The concentration and purity of the DNA samples were analyzed using a Nanodrop spectrophotometer (Thermo Fisher Scientific, Inc., Madison, WI, USA). The V4-V5 hypervariable region (515F-926R) of the 16S rRNA gene was amplified using the primers: 5′-GTGYCAGCMGCCGCGGTAA-3′ and 5′-CCGYCAATTYMTTTRAGTTT-3′ [26] and paired-end sequenced (2 × 250) on the Illumina MiSeq platform.

The paired-end reads were quality-filtered, assembled, and trimmed as described previously [27]. The trimmed sequences were clustered into operational taxonomic units (OTUs) at ≥ 97% sequence similarity using Uclust in QIIME [28]. Subsequently, the OTUs were taxonomically assigned using the Silva 16S rRNA databases (SSU132; https://www.arbsilva.de/) at a confidence threshold of 80%.

### Statistical analysis

The duplicate measurements (i.e. VFA and CH_4_) of individual goats within each sampling week were averaged as one replicate for the statistical analysis. All data were analyzed as a repeated measures ANOVA using the PROC MIXED program in SAS 9.2 (SAS Institute Inc., Cary, NC, USA). The statistical model included NPD, FUM, week, and NPD × FUM, NPD × week, FUM × week, and NPD × FUM × week. interactions as fixed effects, and goat and block as random errors. Sampling week was treated as a repeated measure and goat as a subject. The most desirable covariance structure (unstructured, compound symmetric, and first-order autoregressive) for analysis was determined according to the smallest Bayesian information criterion [23, 29]. When there was a treatment × week. interaction, differences among treatments at each sampling week were reanalyzed using the MIXED procedure with NPD, FUM and NPD × FUM interaction as fixed factors, and block as a random error. When there was an NPD × FUM interaction, Tukey’s multiple comparison test was used to assess differences among treatment means.

The alpha diversity of the samples was estimated using the abundance-based coverage (ACE) estimators, Shannon diversity index, and observed OTUs. Beta diversity of the samples was computed using principal coordinates analysis (PCoA) based on Bray-Curtis dissimilarity [30] in R v.3.6.3 (http://www.R-project.org). Permutational multivariate analysis of variance was performed using the ANOSIM function in the R package vegan to compare the statistical difference in microbial composition across the experimental periods and between treatments.

Statistical significance was declared at *P* < 0.05, while tendency was declared at 0.05 ≤ *P* < 0.10.

## Results

### Methane production and lactation performance

**Fig. 1:**
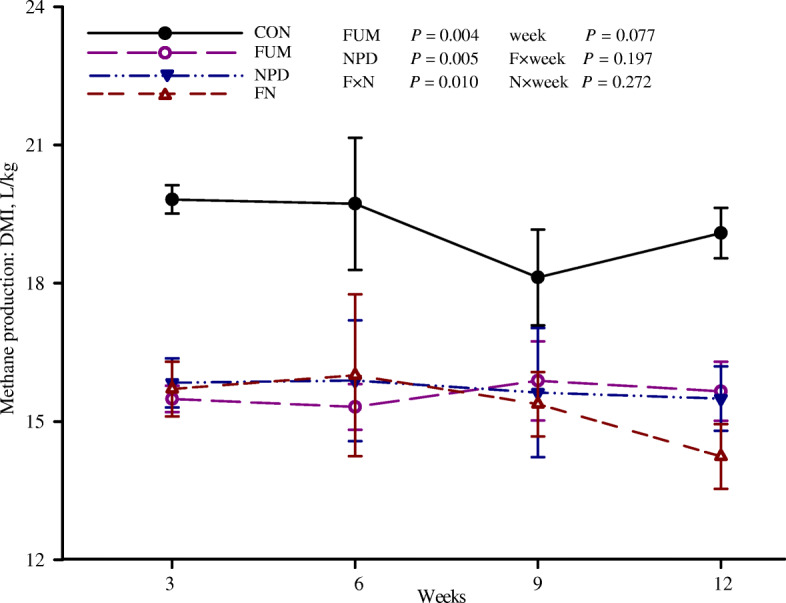
Dynamic and combined effects of fumaric acid (FUM) and N-[2-(nitrooxy)ethyl]-3-pyridinecarboxamide (NPD) on methane yield (L/kg DMI, mean ± standard error) in the dairy goats. *CON*, Control; *FN*, FUM + NPD

The persistent and combined effects of FUM and NPD supplementation on CH_4_ production and milk parameters are shown in Table 2 and Fig. 1. Both FUM and NPD supplementation persistently inhibited (*P* < 0.05) the CH_4_ emissions in goats either expressed as L/d (by 19.1% and 13.4%, respectively) or as L/kg DMI (by 18.8% and 18.1%, respectively) without influencing DMI. A negative interaction (*P* = 0.01) was observed between FUM and NPD in CH_4_ yield (L/kg DMI). The NPD supplementation increased (*P* < 0.05) the DMP, improved feed conversion efficiency expressed as DMP/DMI, and tended to increase the daily FCM production and FCM/DMI, but it decreased (*P* < 0.05) the fat and protein content of the milk without changing milk fat and protein yields. NPD by time interaction was detected for milk protein content (*P* = 0.029), decreasing milk protein content to a greater extent over time (− 9.1% at weeks 3 vs. -20.8% at weeks 9). FUM supplementation had no effects on DMP but decreased (*P* = 0.008) milk fat content and tended to decrease (*P* = 0.065) daily fat yield. In addition, most of the milk parameters changed over time, with DMP (*P* = 0.01) and lactose content (*P* = 0.06) decreasing, whereas fat and protein contents increasing (*P* < 0.01) over time. The time-dependent observations are in line with data reported by Waite et al. [31].

**Table 2:** Effects of the dietary treatments on feed intake, milk performance and methane production of the dairy goats

Item	Treatment^1^				SEM	*P*-value					
	CON	FUM	NPD	FN		Week	FUM	NPD	F × N	F × week	N × week
DMI, kg	1.70	1.70	1.81	1.70	0.080	0.005	0.465	0.511	0.495	0.190	0.958
DMP, kg	1.24	1.29	1.61	1.52	0.127	0.010	0.887	0.035	0.607	0.736	0.235
FCM, kg	1.30	1.19	1.59	1.37	0.125	0.207	0.213	0.085	0.662	0.569	0.259
DMP/DMI	0.73	0.74	0.89	0.90	0.063	0.001	0.862	0.021	0.974	0.915	0.128
FCM/DMI	0.76	0.69	0.87	0.81	0.065	0.046	0.310	0.091	0.912	0.713	0.160
NEL, MJ/d	3.77	3.41	4.45	3.84	0.349	0.171	0.183	0.131	0.724	0.628	0.213
Milk composition, %											
Fat	3.90	3.24	3.41	2.91	0.189	0.001	0.008	0.045	0.683	0.505	0.208
Protein	3.87	3.65	3.26	3.20	0.168	0.001	0.412	0.007	0.633	0.963	0.029
Lactose	4.15	3.97	4.10	3.97	0.128	0.062	0.248	0.825	0.842	0.736	0.239
MUN, mg/L	3.92	3.89	4.03	3.50	0.172	0.157	0.125	0.435	0.161	0.238	0.997
Milk composition yield, g/d											
Fat	46.9	39.1	55.0	43.8	4.78	0.420	0.065	0.202	0.726	0.423	0.307
Protein	46.0	43.7	52.2	48.4	3.57	0.005	0.409	0.152	0.839	0.758	0.258
Lactose	52.0	52.0	65.7	60.3	0.91	0.006	0.642	0.076	0.651	0.950	0.115
Methane emissions											
CH_4_, L/d	32.7^a^	26.4^b^	28.3^b^	25.9^b^	0.91	0.031	0.030	0.036	0.081	0.142	0.342
CH_4_, MJ/d	1.30^a^	1.05^a^	1.12^a^	1.03^a^	0.036	0.031	0.030	0.036	0.081	0.142	0.342
CH_4_/DMI, L/kg	19.2^a^	15.6^b^	15.7^b^	15.3^b^	0.43	0.077	0.004	0.005	0.010	0.197	0.272
CH_4_/FCM, L/kg	26.4	23.7	18.0	19.0	2.56	0.296	0.750	0.043	0.512	0.845	0.094

^1^Means by treatment was the pooled data from goats at weeks 3 and 9, *n* = 3 for the measurements related to CH_4_ and *n* = 6 for the others

^a,b^Means with different superscripts within a row differ (*P* < 0.05). The *P*-values for all the F × N × week. interactions were higher than 0.05, and they were not listed in the table

*CON* Control, *FUM* Fumaric acid, *NPD* N-[2-(nitrooxy)ethyl]-3-pyridinecarboxamide, *FN* FUM+ NPD, *DMI* Dry matter intake, *DMP* Daily milk production, *FCM* Fat corrected milk, *MUN* Milk urea nitrogen, *NEL* Net energy for lactation, *SEM* Standard error of means

### Apparent total tract digestibility and energy balance

The apparent total tract digestibility of nutrients (DM, NDF, ADF, and CP) was not affected by FUM or NPD (Table 3). No change in GE, DE, ME, daily BW change, or energy loss in feces and urine were observed, either. The loss of energy as CH_4_ relative to total GE intake was decreased (*P* < 0.01) by both FUM and NPD, and a negative interaction occurred between these two inhibitors.

**Table 3:** Effects of the dietary treatments on the dietary apparent nutrient digestibility and energy balance of the dairy goats

Item	Treatment^1^				SEM	*P*-value		
	CON	FUM	NPD	FN		FUM	NPD	F × N
Apparent nutrient digestibility, %								
DM	63.8	64.3	63.8	64.0	0.76	0.648	0.792	0.865
NDF	42.0	42.1	41.4	41.9	1.33	0.802	0.777	0.901
ADF	37.7	40.1	38.6	39.5	1.22	0.191	0.900	0.566
CP	82.0	81.8	82.1	81.1	0.56	0.291	0.611	0.497
Energy intake, MJ/d								
GE	28.8	28.5	30.6	28.2	1.37	0.355	0.578	0.469
DE	18.7	18.5	19.8	18.3	0.86	0.373	0.591	0.472
ME	16.5	16.3	17.8	16.2	0.76	0.310	0.457	0.400
Energy loss, MJ/d								
Faeces	10.1	10.0	10.8	9.9	0.57	0.374	0.594	0.508
Urine	0.90	1.14	0.87	1.08	0.182	0.239	0.818	0.947
Methane	1.30	1.06	1.14	1.04	0.041	0.007	0.066	0.134
Energy retention								
NEL, MJ/d	3.87	3.46	4.47	3.79	0.328	0.116	0.179	0.694
BW change, g/d	47.5	43.0	62.5	52.1	8.32	0.383	0.167	0.731
Utilisation of gross energy, %								
DE/GE	64.9	65.3	64.7	65.0	0.74	0.651	0.756	0.920
ME/GE	57.1	57.5	58.1	57.4	1.04	0.902	0.675	0.627
Milk/GE	13.5	12.0	14.6	13.5	1.00	0.208	0.223	0.853
CH_4_/GE	4.52^a^	3.73^b^	3.75^b^	3.70^b^	0.072	0.001	0.001	0.002

^1^Means by treatment was the pooled data from goats at weeks 3 and 9, *n* = 3 for the measurements related to CH_4_ and *n* = 6 for the others

^a,b^Means in the same row with different superscripts differ significantly (*P* < 0.05). The *P*-values for all the F × N × week. interactions were higher than 0.05, and they were not listed in the table

*CON* Control, *FUM* Fumaric acid, *NPD* N-[2-(nitrooxy)ethyl]-3-pyridinecarboxamide, *FN* FUM+ NPD, *BW* Body weight, *GE* Gross energy, *DE* Digestible energy, *NEL* Net energy for lactation, *ME* Metabolizable energy, *SEM* Standard error of means

### Rumen fermentation parameters and bacterial community

The NPD supplementation did not affect any of the measured parameters of rumen fermentation (Table 4). FUM supplementation increased the molar proportion of rumen propionate (*P* = 0.006) but decreased the rumen butyrate proportion (*P* = 0.002), A:P ratio (*P* = 0.018), VFA hydrogen ratio (*P* = 0.005) and the concentrations of fumarate (*P* = 0.003) and succinate (*P* = 0.025). FUM supplementation did not affect rumen total concentration of VFA, pH or the concentration of lactate.

**Table 4:** Effects of the dietary treatments on ruminal fermentation parameters and alpha diversity of microbial community

Item	Treatment^1^				SEM	*P*-value					
	CON	FUM	NPD	FN		Week	FUM	NPD	F × N	F × week	N × week
pH	6.39	6.50	6.46	6.39	0.037	0.001	0.661	0.561	0.033	0.943	0.659
Total VFA production, mmol/L	79.2	75.8	77.5	77.2	3.07	0.001	0.555	0.978	0.624	0.624	0.534
Individual VFA molar proportion, %											
Acetate	65.7	66.4	65.4	65.3	0.46	0.001	0.541	0.142	0.424	0.620	0.088
Propionate	18.1	18.9	17.8	19.6	0.40	0.164	0.006	0.525	0.222	0.394	0.627
Butyrate	12.7	11.5	13.1	11.5	0.37	0.001	0.002	0.603	0.629	0.940	0.196
Valerate	1.13	1.12	1.19	1.21	0.795	0.771	0.795	0.077	0.641	0.923	0.534
A:P	3.67	3.54	3.69	3.34	0.089	0.098	0.018	0.307	0.243	0.367	0.479
VFA hydrogen ratio	8.25	7.84	8.29	7.41	0.196	0.299	0.005	0.327	0.238	0.415	0.619
Fumarate, mmol/L	0.10	0.07	0.10	0.08	0.007	0.001	0.003	0.98	0.451	0.431	0.582
Succinate, mmol/L	2.54	2.02	2.39	1.80	0.225	0.008	0.025	0.425	0.880	0.458	0.445
Lactate, mmol/L	1.14	0.82	0.79	0.78	0.132	0.001	0.226	0.165	0.241	0.752	0.222
Relative abundances of fumarate-utilizing bacteria, %											
*Prevotella ruminicola*	9.50	9.52	8.83	9.70	0.772	0.089	0.574	0.760	0.591	0.724	0.831
*Fibrobacter succinogenes*	1.51	1.67	1.71	1.32	0.184	0.054	0.530	0.687	0.162	0.510	0.845
*Selenomonas ruminantium*	0.18	0.23	0.20	0.30	0.035	0.001	0.059	0.192	0.461	0.187	0.759
Alpha diversity of microbial community											
Observed OTUs	2786	2956	2756	2776	96.8	0.442	0.345	0.297	0.454	0.392	0.883
ACE	5239	5528	5212	5160	171.3	0.027	0.503	0.273	0.340	0.725	0.987
Shannon	8.08	8.07	7.99	8.17	0.089	0.226	0.352	0.954	0.323	0.354	0.693

^1^Means by treatment was the pooled data from goats at weeks 3, 6, 9 and 12, *n* = 5 for the microbial measurements and *n* = 6 for the others

The *P*-values for all the F × N × week. interactions were higher than 0.05, and they were not listed in the table

*CON* Control, *FUM* Fumaric acid, *NPD* N-[2-(nitrooxy)ethyl]-3-pyridinecarboxamide, *FN* FUM+ NPD, *A:P* Acetate: propionate ratio, *SEM* Standard error of means

**Fig. 2:**
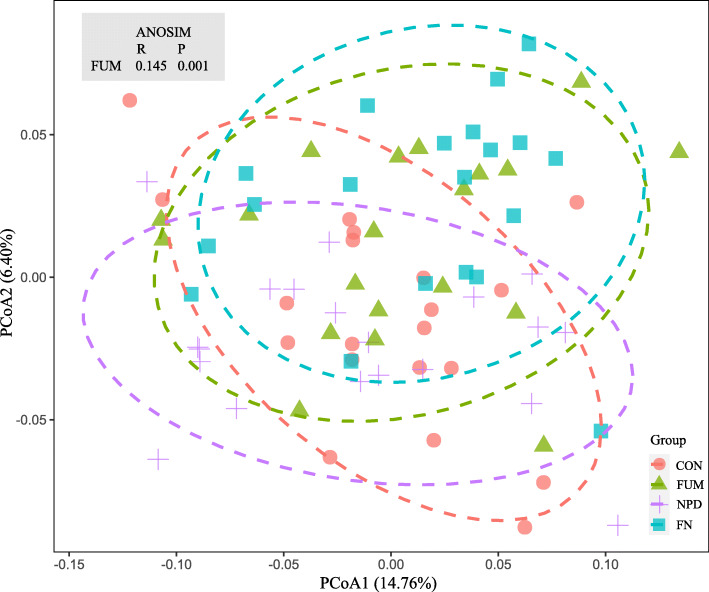
Principal coordinates analysis (PCoA) of ruminal bacterial community based on Bray-Curtis dissimilarity among treatments. The ellipses around each treatment group are based on 80% confidence. *CON*, Control; *FUM*, Fumaric acid; *NPD*, N-[2-(nitrooxy)ethyl]-3-pyridinecarboxamide; *FN*, FUM + NPD

After concatenation and quality filtering, a total of 3.32 M sequences (41,462 per sample) were obtained from the 80 rumen samples. The OTUs were assigned to 22 phyla, 37 classes, 59 orders, 66 families, and 72 genera. At the phylum level, Bacteroidetes (64.7%), Firmicutes (19.4%) and Proteobacteria (6.5%) were predominant. The NPD supplementation did not affect bacterial community composition or diversity (Table 5 and Fig. 2). The bacterial community structure in the animals fed FUM differed from that of the other (Bray-Curtis R_ANOSIM_ = 0.145, *P* = 0.001), particularly changed the structure of the phylum Firmicutes. Within the phylum Firmicutes, the relative abundances of the genera *Ruminococcus*, *Succiniclasticum*, *Clostridium* and *Shuttleworthia* were increased (*P* < 0.05) by FUM, and the genera *Coprococcus* and *Selenomonas* tended to gain higher relative abundance. On the other hand, the genera *Oscillospira* and *RFN20* decreased their relative abundance (*P* < 0.05).

**Table 5:** Effects of the dietary treatments on relative abundances of ruminal dominant bacterial genus (> 0.1%), %

Item	Treatment^1^				SEM	*P*-value					
	CON	FUM	NPD	FN		Week	FUM	NPD	F × N	F × week	N × week
Bacteroidetes, mean = 64.7%											
Prevotella	37.4	36.5	38.3	36.3	0.95	0.166	0.147	0.719	0.571	0.857	0.791
YRC22	2.25	1.96	2.19	1.95	0.207	0.332	0.221	0.871	0.905	0.837	0.791
CF231	1.42^b^	1.79^a^	1.77^a^	1.79^a^	0.098	0.953	0.069	0.100	0.098	0.339	0.704
BF311	0.12	0.15	0.17	0.17	0.016	0.669	0.452	0.076	0.313	0.213	0.635
Paludibacter	0.08	0.28	0.15	0.08	0.089	0.161	0.521	0.482	0.161	0.135	0.361
[Prevotella]	0.11	0.09	0.11	0.14	0.017	0.435	0.656	0.223	0.236	0.506	0.471
Firmicutes, mean = 19.4%											
Ruminococcus	1.83	1.94	1.67	2.05	0.102	0.001	0.034	0.811	0.184	0.891	0.749
Succiniclasticum	1.32	1.52	1.16	1.48	0.099	0.043	0.022	0.320	0.579	0.890	0.481
Oscillospira	1.38	0.97	1.72	0.77	0.198	0.199	0.005	0.726	0.209	0.932	0.252
Coprococcus	1.12	1.22	1.05	1.43	0.120	0.524	0.071	0.580	0.243	0.061	0.683
02d06	0.75	0.80	0.73	0.89	0.073	< 0.001	0.185	0.673	0.496	0.808	0.578
Butyrivibrio	0.79	0.86	0.66	0.83	0.079	0.005	0.146	0.310	0.575	0.158	0.480
RFN20	0.84	0.56	0.79	0.53	0.100	0.266	0.020	0.696	0.906	0.984	0.231
Clostridium	0.46	0.52	0.42	0.50	0.029	0.005	0.038	0.231	0.782	0.227	0.869
Selenomonas	0.23	0.29	0.24	0.38	0.048	0.001	0.070	0.354	0.380	0.137	0.815
Moryella	0.24	0.23	0.23	0.27	0.023	< 0.001	0.672	0.477	0.342	0.331	0.662
Shuttleworthia	0.13^b^	0.14^b^	0.10^b^	0.19^a^	0.015	0.062	0.004	0.470	0.011	0.065	0.713
Proteobacteria, mean = 6.5%											
Ruminobacter	1.67	1.76	1.50	1.14	0.223	0.828	0.548	0.102	0.342	0.097	0.875
Succinivibrio	0.43	0.55	0.63	0.35	0.144	0.006	0.578	0.987	0.189	0.340	0.849
Tenericutes, mean = 2.6%											
Anaeroplasma	0.53	0.39	0.41	0.43	0.049	< 0.001	0.216	0.397	0.150	0.142	0.007
Fibrobacteres, mean = 1.6%											
Fibrobacteres	1.55	1.70	1.71	1.32	0.180	0.024	0.520	0.553	0.160	0.508	0.888
Synergistetes, mean = 0.6%											
TG5	0.80	0.35	0.63	0.30	0.163	0.007	0.035	0.501	0.743	0.037	0.089

^1^Means by treatment was the pooled data from 5 goats at weeks 3, 6, 9 and 12

^a,b^Means with different superscripts within a row differ (*P* < 0.05). The P-values for all the F × N × week. interactions were higher than 0.05, and they were not listed in the table

*CON* Control, *FUM* Fumaric acid, *NPD* N-[2-(nitrooxy)ethyl]-3-pyridinecarboxamide, *FN* FUM+ NPD

### Serum total antioxidant capacity

Supplementation with NPD increased the activity of serum T-AOC and tended to increase the activity of SOD (*P* = 0.053) (Table 6). The FUM supplementation decreased the concentration of MDA (*P* = 0.031) and increased the activity of T-AOC in the serum. An interaction (*P* = 0.007) between FUM and NPD was detected for the activity of T-AOC.

**Table 6:** Effect of dietary treatments on serum antioxidant capacity of the dairy goats

Item	Treatment^1^				SEM	*P*-value					
	CON	FUM	NPD	F × N		Week	FUM	NPD	F × N	F × week	N × week
T-AOC, U/mL	3.54^b^	4.55^a^	4.88^a^	4.41^a^	0.235	0.001	0.269	0.021	0.007	0.644	0.565
GSH-Px, U/mL	286	269	252	276	11.8	0.001	0.769	0.273	0.105	0.898	0.428
SOD, U/mL	65.8	67.5	68.5	71.4	1.59	0.001	0.167	0.053	0.691	0.227	0.570
MDA, μmol/L	1.05	0.79	0.85	0.81	0.069	0.005	0.031	0.248	0.181	0.479	0.874

^1^Means by treatment was the pooled data from 6 goats at 3, 6, 9 and 12 weeks.

^a,b^Means with different superscripts within a row differ significantly (*P* < 0.05). The *P*-values for all the F × N × week. interactions were higher than 0.05, and they were not listed in the table

*CON* Control, *FUM* Fumaric acid, *NPD* N-[2-(nitrooxy)ethyl]-3-pyridinecarboxamide, *FN* FUM + NPD, *MDA* Malondialdehyde, *GSH-Px* Glutathione peroxidase, *SOD* Superoxide dismutase, *T-AOC* Total antioxidant capacity, *SEM* Standard error of means

## Discussion

### Long-term effects of NPD on daily CH_4_ production

To our knowledge, no *in vivo* studies have been published on the effects of NPD in CH_4_ production since its first evaluation via *in vitro* fermentation [8]. Consistent with the effects of 3-NOP [6, 10], a most researched nitrooxy compound, supplementation with NPD resulted in a reduction (by 18.1%) in CH_4_ emissions in dairy goats, and the inhibitory effect persisted throughout the 12-week treatment. A recent meta-analysis based on dairy and beef cattle trials showed that an average dose of 123 mg 3-NOP per kilogram of feed dry matter (FDM) reduced CH_4_ emissions by 29.3 ± 5.63% [32], which is higher than our observation (by 18.1%). The extent of methane inhibition by 3-NOP is dependent on the dose and administration technique [32, 33]. The dose of NPD in the current study was 276 mg per kilogram of FDM, equivalent to 158 mg of 3-NOP/FDM based on the molecular weight and mole of the nitrooxy group. At a similar supply dose (150 mg/kg FDM), the extent of CH_4_ emission reductions (− 18.1%) by NPD in the present study was also much lower than that (− 36%) by 3-NOP [34]. Considering the high oxidability of nitrooxy groups and the low redox potential of the rumen environment, nitrooxy groups can be reduced in the rumen [7], and it has been shown that the antimethanogenic effects of 3-NOP are the highest within 6 h after feeding [10]. When NOP was dosed into the rumen of dairy cattle, Reynolds et al. [35] found that CH_4_ production dropped substantially immediately after dosing, but the effect was only sustained for 1 to 2 h. Therefore, one possible explanation for the lower CH_4_ emission reduction by NPD observed in this study could be the administration technique of top-dressing on the TMR, which could not allow for continual consumption and decreasing CH_4_ emissions throughout the day as mixed inclusion of 3-NOP in the TMR [32, 34]. This premise is consistent with the greater reduction in CH_4_ emissions (59%) observed when 280 mg of 3-NOP/kg FDM was mixed with beef cattle TMR [36] than that (33%) when up to 345 mg of 3-NOP/kg FDM was top-dressed on the same background diet [37]. Differences in molecular structure may also be responsible for the different CH_4_ mitigation potentials between 3-NOP and NPD [8], and future studies are needed to compare the two nitrooxy compared both *in vivo* and *in vitro* to determine their relative efficacy.

### Long-term effects of FUM on CH_4_ production and ruminal VFA profiles

The persistence of the CH_4_-decreasing effect is an important criterion in evaluating the potential of CH_4_ emission reduction strategies [1], and to our knowledge, no studies have been reported to evaluate this criterion for FUM. Supplementation of the diet with FUM enhanced rumen propionate fermentation accompanied by a decrease in CH_4_ emissions, consistent with the results of previous studies [16, 17, 38], and these responses persisted over the whole 12-week treatment period. Theoretically, conversion of all 34 g FUM (0.29 mol) to propionate could potentially reduce daily CH_4_ yield by 1.80 L [17], which is much lower than the reduction (on average of 6.25 L) observed in the current study, supporting the earlier theory that the mechanism of FUM action in CH_4_ suppression was not only attributable to its function as an H-acceptor [17, 38]. *Prevotella ruminicola*, *Fibrobacter succinogenes* and *Selenomonas ruminantium* have been recognized as rumen fumarate-utilizing bacteria [39, 40], but only the relative abundances of *Selenomonas ruminantium* tended to be more abundant (*P* = 0.059) in the animals fed with FUM, which is in agreement with the previous findings in sheep [16]. Instead of increase, the concentrations of rumen fumarate and succinate decreased in the goats fed FUM compared with the goats fed CON, probably due to the substrate stimulatory effects of FUM on fumarate-utilizing bacteria [41], and thus increasing the ruminal activity of the succinate-propionate metabolic pathway.

### Combined effects of FUM and NPD on ruminal hydrogen flow potential

Hydrogen is an important fermentation intermediate in the rumen [42], mainly originating from the acetate- and butyrate-forming pathways. The produced hydrogen is primarily removed via methanogenesis and propionogenesis [13]. The inhibition of methanogenesis is expected to redirect excess hydrogen to propionate synthesis [14, 43]. However, the ruminal proportion of propionate does not always increase when methanogenesis is inhibited by 3-NOP [10, 44], consistent with our results. An increase (by 48- to 100-fold) in eructated gaseous hydrogen is commonly observed *in vitro* or* in vivo* with 3-NOP supplementation [6, 8–10], suggesting that the efficiency of hydrogen capture was lower when CH_4_ production was inhibited by 3-NOP [12, 45]. Only 54.3% of the hydrogen spared from methanogenesis was diverted to alternate hydrogen-sinks *in vitro* [15], and 31% of the spared hydrogen was released as gas in beef cattle [45]. Consequently, the negative interaction between NPD and FUM was expected as the hydrogen was enough for both methanogenesis and propionogenesis, resulting in their competitive relationship disappearing when both NPD and FUM were supplemented in goats.

If 4 mol of H_2_ and 1 mol of CO_2_ are required to yield 1 mol of CH_4_, the energy loss associated with eructated gaseous H_2_ is 27% higher than that of CH_4_. Moreover, the global warming potential of eructated gaseous H_2_ is close to that of converted CH_4_ (4 × 5.8: 25, on a CO_2_-equivalent basis) [11, 12]. In addition, the volume of eructated gaseous H_2_ and CO_2_ is 4-fold higher than that of converted CH_4_, resulting in a risk of rumen flatulence. Taken together, an increase in eructated gaseous H_2_ partly offsets the advantages of energy savings and reduced environmental concerns by CH_4_ mitigation. Therefore, it is desirable for the spared H_2_ to be efficiently diverted to nutritionally beneficial sinks, such as propionate [45]. In this study, FUM showed greater responses in propionate increase when supplemented in combination with NPD than alone (by 10.2% vs. 4.4%), suggesting FUM diverted more hydrogen towards propionate synthesis when supplemented in combination. Because NPD, FUM, and their combination resulted in similar CH_4_ emissions, it indicates that the release of gaseous H_2_ was lower from the animals fed both NPD and FUM than those fed NPD alone. Similar results have also been observed in beef cattle when supplementation of 3-NOP was combined with monensin [45], with the combination increasing more propionate proportion than supplementing monensin alone (by 29.8% vs. 11.6%), and the combination decreasing H_2_ emissions by 79.7% compared with supplementing 3-NOP alone.

### Effects of NPD on lactation performance

Beyond expectation, NPD increased DMP by 29.8%, while decreasing milk fat content by − 12.6% and milk protein content by − 15.8%, without changes in nutrient digestibility, rumen VFA profiles, or daily BW gain. Although CH_4_ suppression in the rumen has the potential to improve energy efficiency [45], the mean decrease in CH_4_ energy of 0.18 MJ/d per goat by NPD would only convert to 0.04 MJ/d of milk NEL [10, 21], which is much lower than the actual improvement of milk NEL (0.68 MJ) observed in the goats. The NPD dose used in the current study was about 13 mg/kg/d, much higher than the level (1 mg/kg/d) that increased blood flow in mice [19]. A possible explanation for the unexpected improvement of milk NEL by DMP might be that some of the NPD escaped from rumen fermentation and was absorbed by the gut, ultimately exerting its bioactive function, such as increasing blood flow. Indeed, several studies have shown that blood flow was positively associated with milk production and uptake of milk precursors [46–48]. However, the blood flow and blood NPD content were not measured in this study. To support this explanation, we measured another bioactive function of NPD, enhancing antioxidant capacity [49, 50], which is consistent with our observations. Therefore, the side effects of this nitrooxy compound on animal health and its residues in animal products need further evaluation before it can be used as an animal feed additive.

### Effects of FUM on lactation performance

Supplementation with FUM did not affect DMP, although it was accompanied by a series of positive effects, such as inhibiting CH_4_ production, increasing propionate proportion and the relative abundances of rumen cellulolytic bacterial genera (e.g., *Ruminococcus* and *Clostridium*). The null effect of FUM on DMP is consistent with that observed in dairy cows receiving FUM in previous studies [38, 51]. On the other hand, the inclusion of FUM decreased milk fat content and tended to decrease milk fat yield, without changing other milk components, which are close to the classical characteristics of diet-induced milk fat depression [52]. Similar results were also observed in dairy cows receiving 600 g FUM supplementation per day [51]. The decreased rumen butyrate proportion and acetate-to-propionate ratio in response to FUM might be partially responsible for the lower milk fat because acetate and butyrate are important precursors for the de novo synthesis of milk fatty acids [53, 54]. However, more recent experiments revealed that shifts in the rumen VFA profile do not seem to be a major cause of milk fat depression [52]. Diets known to induce milk fat depression were associated with rumen unsaturated fatty acid biohydrogenation [52], which will be explore in our further research.

## Conclusions

Using lactating dairy goats as a model, we evaluated the effects of NPD as a direct methanogenesis inhibitor, fumarate as an alternative hydrogen sink, and their combination on CH_4_ production, rumen fermentation, and lactation performance over 12 weeks. Both NPD and FUM persistently inhibited CH_4_ emissions without negative influences on DMI or nutrient digestibility. The hydrogen spared from the inhibited methanogenesis by NPD was more likely used for propionate synthesis rather than being eructated as gas when FUM was also supplemented. However, NPD and other nitrooxy compounds need to be further evaluated for their side effects on animal health and their residues in animal products before they can be used as animal feed additives.

## Data Availability

The original sequence data had been deposited to NCBI with Bioproject accession no. PRJNA703427.

## Abbreviations

A:P: Acetate to propionate ratio
ADF: Acid detergent fiber
BW: Body weight
DE: Digestible energy
DIM: Days in milk
DMP: Daily milk production
DMI: Dry matter intake
FCM: Fat corrected milk
FDM: Feed dry matter
FUM: Fumaric acid
GE: Gross energy
GSH-Px: Glutathione peroxidase
MDA: Malondialdehyde
ME: Metabolizable energy
MUN: Milk urea nitrogen
NEL: Net energy for lactation
NDF: Neutral detergent fiber
NPD: N-[2-(nitrooxy)ethyl]-3-pyridinecarboxamide
VFA: Volatile fatty acids
SMD: Standardized mean difference
SOD: Superoxide dismutase
T-AOC: Total antioxidant capacity
